# Role of Lipoprotein(a) and Blood Cells Ratios in Peripheral Artery Disease

**DOI:** 10.3390/ijms26209918

**Published:** 2025-10-12

**Authors:** Alexandra V. Tyurina, Olga I. Afanasieva, Marat V. Ezhov, Narek A. Tmoyan, Tatiana V. Balakhonova, Sergei N. Pokrovsky

**Affiliations:** 1 A.L. Myasnikov Institute of Clinical Cardiology, National Medical Research Center of Cardiology n.a. acad. E.I. Chazov, Ministry of Health of the Russian Federation, Moscow 121552, Russia; ntmoyan@gmail.com (N.A.T.); tvbdoc@gmail.com (T.V.B.); 2 Institute of Experimental Cardiology, National Medical Research Center of Cardiology n.a. acad. E.I. Chazov, Ministry of Health of the Russian Federation, Moscow 121552, Russia; afanasieva.cardio@yandex.ru (O.I.A.); dr.pokrovsky@mail.ru (S.N.P.)

**Keywords:** lipoprotein(a), peripheral arterial disease, monocytes, blood counts, chronic ischemia of the lower limb

## Abstract

Peripheral artery disease (PAD) is a major global health issue. This study investigated the relationship between lipoprotein(a) [Lp(a)], high-density lipoprotein cholesterol (HDL-C) to blood cells ratios, and PAD development. The study included 361 patients categorized into groups based on the presence of stenotic atherosclerosis in lower limb arteries (LLAs) diagnosed via duplex ultrasound. Group 1 (n = 238) had atherosclerosis at the first visit. A second visit involved 281 patients: 158 from Group 1, 32 new diagnoses (Group 2), and 91 with no atherosclerosis at either visit (Group 3). Laboratory analysis included lipid profiles, Lp(a), and complete blood counts, calculating ratios like Lp(a)/HDL-C and monocyte-to-HDL-C ratio (MHR). Showed patients with stenotic atherosclerosis had significantly higher Lp(a) (20.2 vs. 12.1 mg/dL, *p* < 0.01), MHR (0.54 vs. 0.39, *p* = 0.002), and Lp(a)/HDL-C ratios (20.9 vs. 8.8, *p* = 0.003). The combination of monocytes ≥ 0.55 × 10^9^/L and Lp(a) ≥ 30 mg/dL was present in 27% of PAD patients vs. 10% without (*p* < 0.01). Kaplan–Meier analysis indicated that high Lp(a) levels led to chronic limb ischemia 9.5 years earlier. Combined assessment of Lp(a) and monocyte-related ratios provides superior predictive value for PAD, suggesting clinical utility for risk stratification and early intervention.

## 1. Introduction

According to data from the Global Burden of Disease Study 2019, published in *The Lancet Global Health* in 2023, peripheral artery disease (PAD) represents a significant global health problem. In 2019, the number of people aged 40 years and older with PAD amounted to 113 million worldwide, which corresponds to a global prevalence of 1.52% [1]. According to the study "Health Disparities in Peripheral Artery Disease”, the total number of patients with PAD in the world exceeds 200 million [2]. Prevalence increases significantly with age; among people aged 80–84 years it reaches 14.9% [1]. According to official statistics, the overall incidence of peripheral artery diseases in the Russian Federation is 406 cases per 100,000 adult population [3].

Among the most promising directions is the evaluation of inflammatory indices, using blood cell counts. The neutrophil-to-lymphocyte ratio (NLR) is considered as a marker of systemic inflammation [4,5]. The lymphocyte-to-monocyte ratio (LMR) reflects the balance of anti-inflammatory and pro-inflammatory processes [6]. The platelet-to-lymphocyte ratio (PLR) characterizes the state of inflammatory activity and hypercoagulation [7]. There is a particular interest presented by combined parameters including high-density lipoprotein cholesterol (HDL-C): the ratio of monocytes to HDL-C [8,9], neutrophils to HDL-C [10], and platelets to HDL-C [11], that integrate inflammatory status and lipid metabolism. Preliminary studies have shown the potential diagnostic and prognostic value of these parameters in cardiovascular diseases [12]. However, a systematic evaluation of their clinical significance in peripheral artery disease has not yet been performed. Moreover, optimal threshold values of indices and their place in patient risk assessments remain undefined.

## 2. Results

### 2.1. Biomarkers of Stenotic Atherosclerosis of Lower Limbs 

All three groups were comparable in age (Table 1). When comparing the groups, a significant predominance of males was observed among patients in whom stenotic atherosclerosis of lower limb arteries was diagnosed at the first visit compared to those in whom atherosclerosis was not detected at either the first or second examination (81.5% vs. 64.8%; *p* < 0.01). The proportion of patients with elevated Lp(a) was significantly higher in the group with stenotic atherosclerosis of lower limbs (43.3%) compared to the group without stenotic atherosclerosis (24.2%), *p* < 0.01. The highest frequency of coronary artery disease was observed in patients with stenotic atherosclerosis of lower limb arteries detected at the first visit (80.7%) compared to patients who had no atherosclerosis initially but were diagnosed during the follow-up period (46.9%) and patients without atherosclerosis at both visits (41.8%) (*p* < 0.001). Lp(a) levels also differed significantly between groups: the median in the group with baseline stenotic atherosclerosis of lower limb arteries was 20.2 mg/dL [IQR: 6.7–88.5], which was higher than in the group without atherosclerosis at both visits −12.1 mg/dL [IQR: 5.2–26.8]; *p* < 0.01.

In forming the study groups, we specifically defined Group 2, in which stenotic atherosclerosis developed between the first and second visits, to identify early predictors and markers of atherosclerotic process progression. This approach is justified by the principles of prospective risk factor analysis and allows the identification of clinical and laboratory parameters that, at the time of initial examination (before manifestation of stenotic atherosclerosis), were predictive of the increased risk of disease development in the short-term.

In patients with stenotic atherosclerosis of lower limb arteries detected at the first visit (Group 1), neutrophil count (4.2 [3.4; 5.4] × 10^9^/L) and CRP concentration were higher (1.1 [0.4; 2.4] mg/L) compared to Group 3 patients, in whom stenotic atherosclerosis of lower limb arteries was not detected either at baseline or during follow-up (neutrophils: 3.6 [3.0; 4.6] × 10^9^/L, *p* = 0.04; CRP: 0.4 [0.1; 0.7] mg/L, *p* = 0.01). Furthermore, patients with baseline atherosclerosis had significantly higher calculated indices: MHR (0.54 [0.40; 0.73] vs. 0.39 [0.30; 0.55], *p* = 0.002) and Lp(a)-to-HDL-C ratio (20.94 [6.19; 71.79] vs. 8.82 [4.41; 21.39], *p* = 0.003) (Table 1).

**Figure 1:**
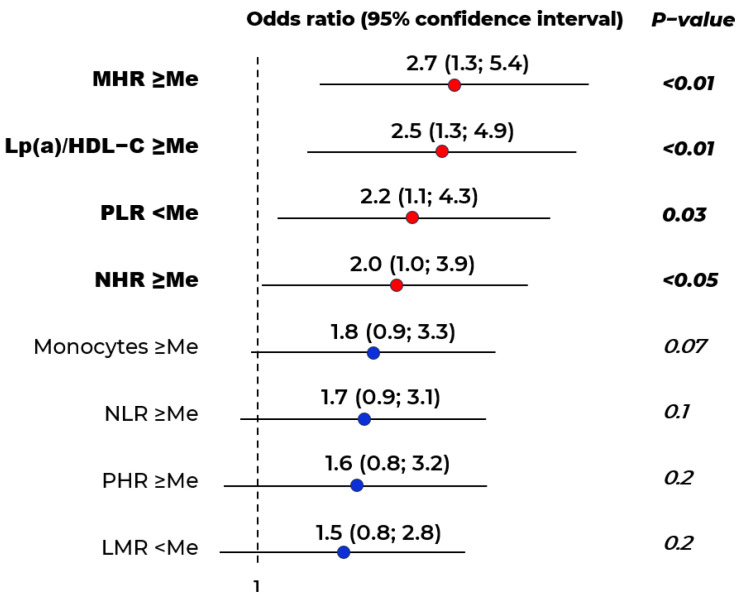
Odds ratios for stenotic atherosclerosis of the lower limb arteries. Me–median. Median values for indicators: monocyte-to-HDL-C ratio (MHR)—0.5; neutrophil–lymphocyte ratio (NLR)—1.79; neutrophil-to-HDL-C ratio (NHR)—3.6; platelet-to-HDL-C ratio (PHR)—203.0; platelet-to-lymphocyte ratio (PLR)—105.7; monocytes—0.55 × 10^9^/L, lymphocyte–monocyte ratio (LMR)—4.0.

Odds ratio analysis revealed that MHR > 0.5 (OR = 2.7, *p* = 0.005), Lp(a)-to-HDL-C ratio > 16.3 (OR = 2.5, *p* < 0.01), NHR > 3.6 (OR = 2.0, *p* < 0.05), and PLR < 105.7 (OR = 2.2, *p* < 0.05) were associated with stenotic atherosclerosis of lower limb arteries at the first visit (Figure 1). After applying Bonferroni correction for multiple comparisons, the only parameter that demonstrated a statistically significant association with stenotic atherosclerosis of lower limb arteries (Group 1) was MHR ≥ median value.

The median monocyte count at the second visit was also 0.55 × 10^9^/L. In patients with monocyte levels below the median (<0.55 × 10^9^/L) and Lp(a) concentration < 30 mg/dL, the relative risk of detecting stenotic atherosclerosis of lower limbs at the second visit (n = 32) was 57% lower (RR = 0.43; 95% CI: 0.18–1.02; *p* < 0.05) than in patients with any other combination of these two parameters (Group 3 served as control).

**Figure 2:**
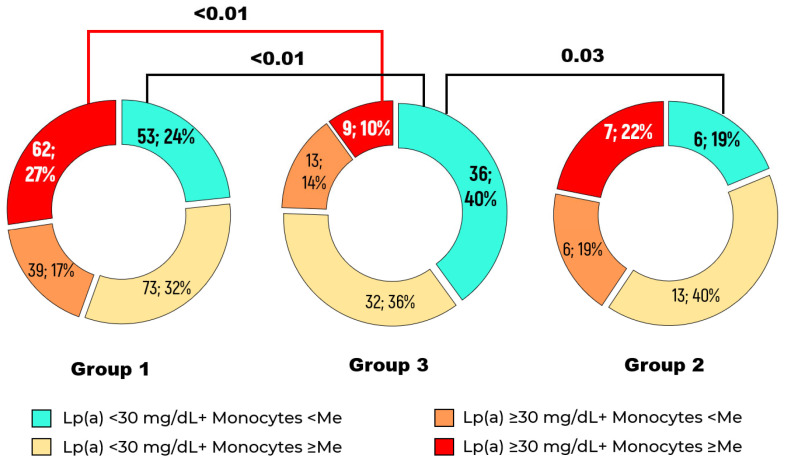
Distribution of patients depending on Lp(a) concentration and absolute monocyte count in Groups 1, 2, and 3. The median monocyte count was 0.55 × 10^9^/L.

The combination of monocyte count ≥ median value of 0.55 × 10^9^/L and Lp(a) level ≥ 30 mg/dL was found in 27% of patients with stenotic atherosclerosis (Group 1) versus 10% of patients without detected stenotic atherosclerosis of lower limb arteries throughout the entire follow-up period (Group 3), *p* < 0.01 (Figure 2). The presence of stenotic atherosclerosis of lower limb arteries was associated with a monocyte count above the median combined with an elevated Lp(a) concentration (OR = 2.5, 95% CI: 1.4–4.5, *p* < 0.01) compared to patients with Lp(a) level < 30 mg/dL and a monocyte count below the median.

**Figure 3:**
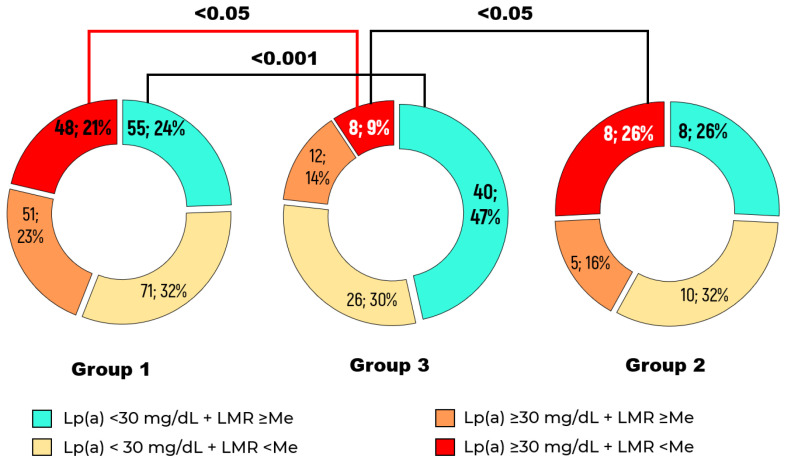
Distribution of patients depending on Lp(a) concentration and lymphocyte–monocyte ratio (LMR) level in Groups 1, 2, and 3. The median LMR was 4.0.

The combination of an LMR value below the median of 4.0 and Lp(a) level ≥ 30 mg/dL was found in 21% of patients with stenotic atherosclerosis versus 9% of patients without detected stenotic atherosclerosis of lower limb arteries throughout the entire follow-up period, *p* < 0.05 (Figure 3). However, the presence of stenotic atherosclerosis of lower limb arteries was not significantly associated with an LMR below the median in the setting of Lp(a) concentration > 30 mg/dL (OR = 1.7, 95% CI: 0.9–3.2, *p* < 0.1) compared to patients with Lp(a) level < 30 mg/dL and LMR above the median distribution.

**Figure 4:**
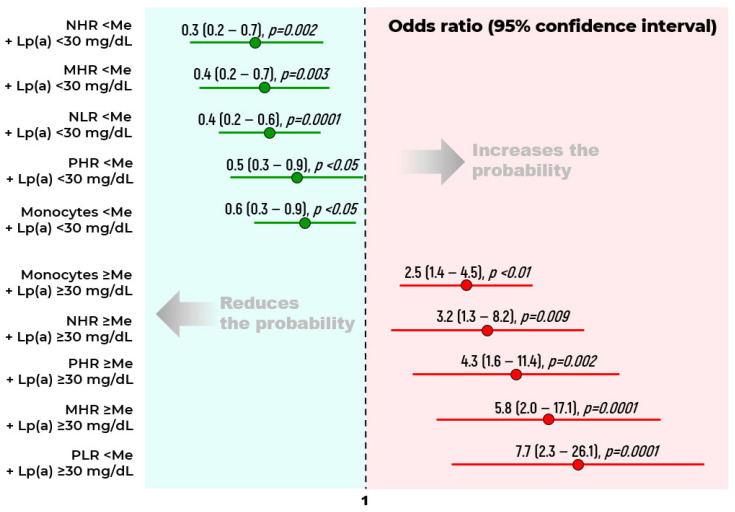
Odds ratios for stenotic atherosclerosis of the lower limb arteries at the first visit. Me—median. Median values for: neutrophil–lymphocyte ratio (NLR)—1.79; monocyte-to-HDL-C ratio (MHR)—0.5; neutrophil-to-HDL-C ratio (NHR)—3.6; platelet-to-HDL-C ratio (PHR)—203.0; platelet-to-lymphocyte ratio (PLR)—105.7; monocytes—0.55 × 10^9^/л. The reference group included all possible combinations of the two parameters except for the investigated combination.

The analysis demonstrates that the combined assessment of Lp(a) levels with several other markers shows a stronger association with atherosclerosis than the evaluation of individual parameters (Figure 4).

The systematic testing of nine different weighted combinations showed that the calculated composite index [0.3 × Lp(a) concentration (mg/dL) + 0.7 × absolute monocyte count (×10^9^/L)] is in the optimal zone for the predictive abilities of stenotic atherosclerosis development in lower limb arteries (AUC = 0.63). Clinical validation demonstrated a high discriminating ability of this calculated index: when using the median (5.3) as the cut-off value, patients with values above the median had 2.0-fold higher odds of developing stenotic peripheral atherosclerosis (OR 2.0, 95% CI: 1.2–3.3, *p* = 0.008).

Multiple logistic regression analysis, simultaneously incorporating monocytes, neutrophils, Lp(a), PLR, LMR, NLR, MHR, PHR, and CRP into the model, revealed that only Lp(a) concentration and the MHR were independent predictors of PAD. The model demonstrated high statistical significance (*p* < 0.0001). Specifically, for every 10 mg/dL increase in Lp(a) concentration, the risk of developing PAD increased by 10% (*p* < 0.001), and for every 0.1-unit increase in the MHR, the likelihood of the disease increased by 30% (*p* = 0.0003). The overall classification accuracy of the model was 71.2%.

The characteristics of the patients at the second visit are shown in Table 2.

When examining parameters at the second visit, the only factor combinations demonstrating statistically significant association with atherosclerosis development at the second visit (Group 2) compared to the control group (Group 3, n = 91) in odds ratio analysis were as follows: a combination of HDL-C level below the median (1.12 mmol/L) and elevated Lp(a) concentration (≥30 mg/dL) (OR = 8.2, 95% CI: 1.5–44.9, *p* = 0.01), as well as a combination of PLR below the median (101.9) and elevated Lp(a) concentration (≥30 mg/dL) (OR = 5.0, 95% CI: 1.3–19.2, *p* = 0.02).

### 2.2. Biomarkers of Chronic Lower Limb Ischemia

**Figure 5:**
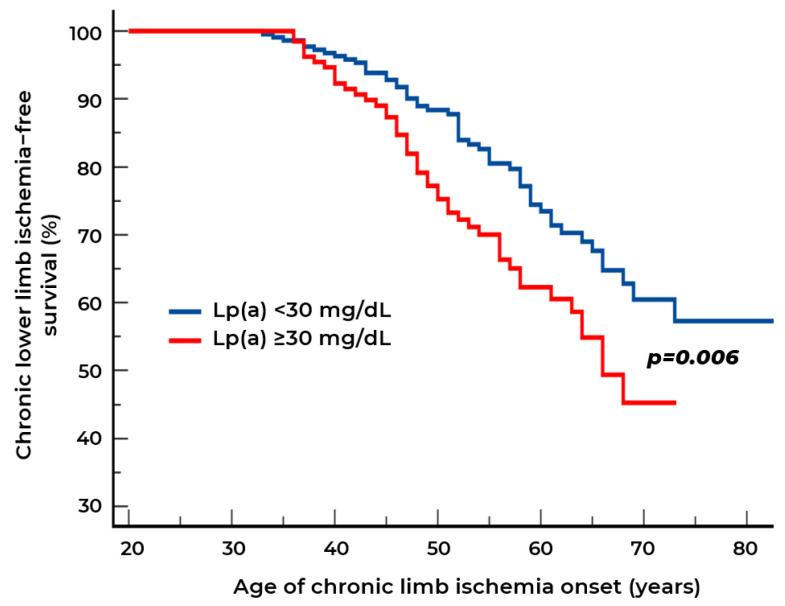
Survival curves for chronic lower limb ischemia (CLLI) in patients with stenotic atherosclerosis of lower extremity arteries according to lipoprotein(a) [Lp(a)] concentration.

Among Groups 1 and 2, CLLI was detected in 101 patients at the second visit. Kaplan–Meier curve analysis revealed that patients with high Lp(a) levels (≥30 mg/dL) develop CLLI significantly earlier than individuals with normal Lp(a) levels. By age 70, survival without CLLI in the high Lp(a) group is approximately 45%, while in the low-level group it is approximately 60% (Figure 5). For the group with Lp(a) ≥ 30 mg/dL, the median age of CLLI onset is approximately 65 years, while for the group with Lp(a) < 30 mg/dL, the curve does not cross the 50% level in the visible portion of the graph, indicating that the median age exceeds 80 years. When calculating the mean value, the difference in age of CLLI development is 9.5 years.

**Figure 6:**
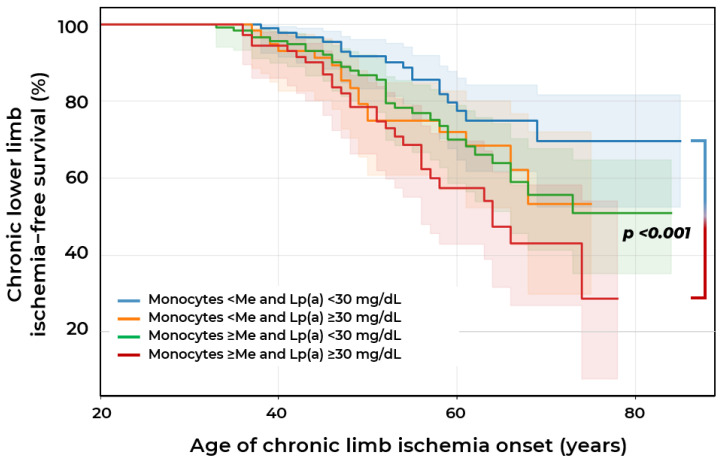
Survival curves for chronic lower limb ischemia (CLLI) in patients with stenotic atherosclerosis of lower extremity arteries according to lipoprotein(a) concentration and monocyte count relative to the median value of 0.55 × 10^9^/L.

In the group of patients with the combination of absolute blood monocyte count above the median (≥0.55 × 10^9^/L) and Lp(a) (≥30 mg/dL), not only was earlier onset of CLLI identified, but also an almost two-fold higher frequency of CLLI occurrence (38.7% vs. 17.9%) compared to the group with lower blood monocyte count (<0.55 × 10^9^/L) and normal Lp(a) level (<30 mg/dL) (*p* < 0.001). This confirms that the combination of these two factors substantially increases the overall risk of CLLI (Figure 6).

**Figure 7:**
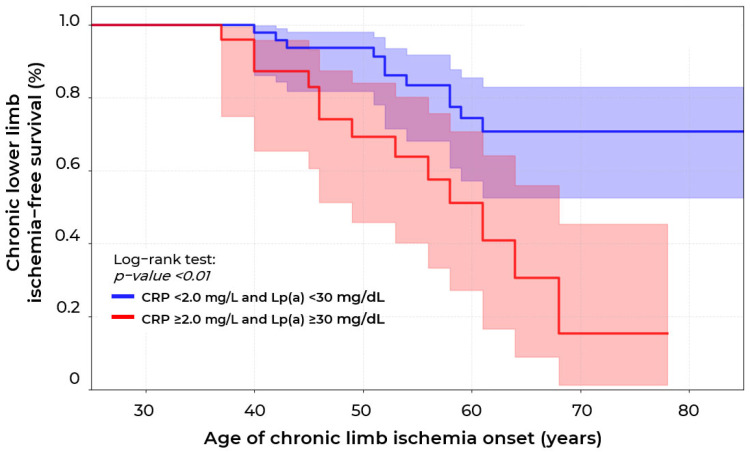
Survival curves for chronic lower limb ischemia (CLLI) in patients with stenotic atherosclerosis of lower extremity arteries according to lipoprotein(a) concentration (≥ and <30 mg/dL) and C-reactive protein (CRP ≥ and <2.0 mg/L).

Kaplan–Meier curve analysis (Figure 7) (n = 136) revealed statistically significant differences in survival without CLLI development between patients with CRP concentration < 2.0 mg/L and Lp(a) < 30 mg/dL compared to patients with CRP concentrations ≥ 2.0 mg/L and Lp(a) ≥ 30 mg/dL (*p* < 0.01, log-rank test). Patients with the combination of elevated CRP and Lp(a) concentrations showed a 2.2-fold higher relative risk of CLLI development (95% CI: 1.1–4.2, *p* = 0.03) compared to patients with CRP < 2.0 mg/L and Lp(a) < 30 mg/dL, with median survival of 63 years versus an unreached median in the group of patients with normal CRP and Lp(a) concentrations. The survival curves demonstrate progressive divergence with age: by 70 years, survival without CLLI in patients with the combination of high CRP and Lp(a) concentrations was only 17.2% versus 67.8% in the low-risk group, corresponding to an absolute risk increase of 26.9% and NNH = 4, indicating that every fourth patient with high CRP and Lp(a) levels is subject to the additional risk of CLLI development.

**Figure 8:**
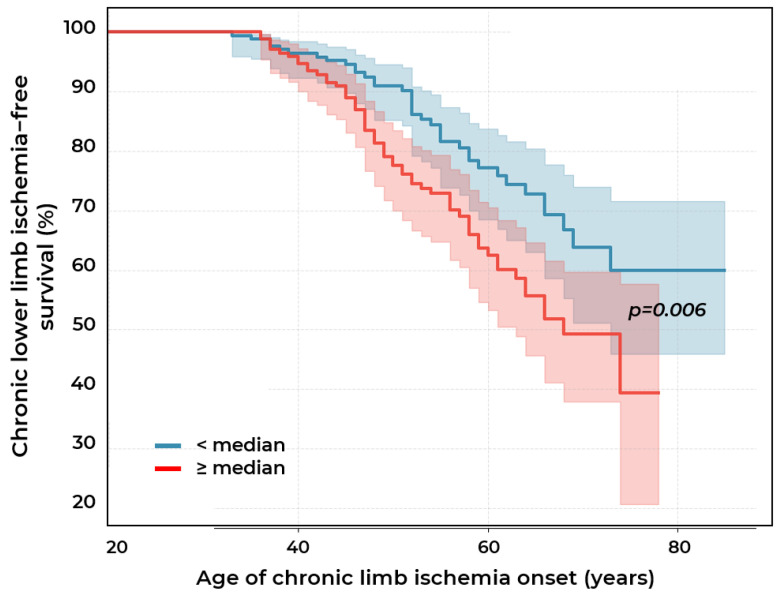
Survival curves for chronic lower limb ischemia (CLLI) in patients with stenotic atherosclerosis of lower extremity arteries according to composite index [0.3 × Lp(a) concentration (mg/dL) + 0.7 × absolute monocyte count (×10^9^/L)] values (<median of 5.3 or ≥median).

Kaplan–Meier survival analysis showed that the median age of CLLI development in the high-risk group (composite index [0.3 × Lp(a) concentration (mg/dL) + 0.7 × absolute monocyte count (×10^9^/L)] ≥ 5.3) was 68 years. Meanwhile, in the low-risk group (index < 5.3), the median was not reached during the observation period, indicating a substantially more favorable prognosis in this group. The probability of absence of CLLI by age 70 was significantly higher among patients with an index < 5.3 compared to the ≥5.3 group (63.8% vs. 49.2%) (Figure 8). Cox regression analysis showed a significantly increased risk of CLLI in patients with index values > 5.3 compared to the ≤5.3 group (HR = 1.8; 95% CI: 1.2–2.7; *p* = 0.005) independent of hypertension, type 2 diabetes, and sex.

## 3. Discussion

We propose that a comparison of Group 2 (in whom PAD developed between the first and second visit) with Group 1 and Group 3 allows the identification of clinical and laboratory parameters that at the time of initial examination (before manifestation of stenosing lesions) were predictive of an increased risk of disease development in the short-term. In our study, we utilized a threshold of 30 mg/dL, which is adopted in Russian clinical guidelines and corresponds to the population under investigation. Furthermore, our previous research has confirmed the association of Lp(a) levels ≥ 30 mg/dL with the development and progression of carotid and coronary artery atherosclerosis [13], as well as with early-onset CAD, providing additional rationale for the selection of this cut-off value.

### 3.1. Absolute Monocyte Count

Our results emphasize the important role of circulating blood monocytes in the development of stenotic atherosclerosis of lower limb arteries. Although circulating blood monocyte count above the median in our study was associated with peripheral atherosclerosis of only borderline significance (OR 1.8; 95% CI 0.9–3.3, *p* = 0.07), the combination of this parameter with higher Lp(a) levels ( ≥ 30 mg/dL) was observed almost three times more frequently in patients with peripheral atherosclerosis than in patients without atherosclerosis (27% vs. 10%, *p* < 0.01), indicating synergism between inflammatory and lipid factors. Moreover, patients who had higher blood monocyte counts with an elevated Lp(a) concentration showed not only earlier onset of CLLI, but also an almost two-fold increase in its frequency (38.7% vs. 17.9%, *p* < 0.001) compared to the group of patients with low values of both parameters (monocytes < 0.55 × 10^9^/L and Lp(a) < 30 mg/dL). The composite calculated index proposed in our study [0.3 × Lp(a) + 0.7 × absolute monocyte count] has the ability to predict stenotic atherosclerosis development in lower limb arteries (AUC = 0.63). Patients with a composite calculated index of 5.3 and above, corresponding to above the median value, had 2.0-fold higher odds of developing stenotic peripheral atherosclerosis (OR 2.0, 95% CI: 1.2–3.3, *p* = 0.008). Furthermore, Kaplan–Meier survival analysis showed that such patients with an index ≥ 5.3 had a median age of CLLI development of 68 years. Conversely, in the group with an index < 5.3, the median was not reached during the entire observation period. A 1.8-fold increased risk of CLLI in patients with higher composite calculated index values, independent of hypertension, type 2 diabetes, and sex, confirms the importance of the combined influence of Lp(a) concentration and circulating blood monocyte count on peripheral atherosclerosis development in lower limbs.

Our findings are consistent with the literature data on the role of monocytes in atherogenesis. In our previous study, patients with both elevated Lp(a) (≥30 mg/dl) and a high monocyte count (>0.55 × 10^9^/l) had 16.8-times higher odds of carotid atherosclerosis progression [14]. Wei et al. showed that median monocytes increase from 0.50 [0.37; 0.62] × 10^9^/L in the group without atherosclerosis to 0.63 [0.48; 0.80] × 10^9^/L in the group with severe stenosis of lower limb arteries [9]. Wildgruber et al., in a study of patients with severe peripheral artery disease, showed an increase in the proportion of the "intermediate" monocyte subpopulation CD14 ++CD16 +, which has enhanced pro-inflammatory activity [15]. The increase in monocyte content in atherosclerosis is explained by their key role in the initiation and progression of the atherosclerotic process. Monocytes migrate into the arterial intima, differentiate into macrophages, absorb modified lipoproteins, and transform into foam cells—the main cellular elements of atherosclerotic plaques [16]. Our study data confirm that absolute circulating blood monocyte count is not only a marker of systemic inflammation but also an active participant in atherogenesis, especially in combination with elevated Lp(a) levels. A combined assessment of these parameters can significantly improve risk stratification and disease course prediction in patients with stenotic atherosclerosis of lower limb arteries.

### 3.2. Monocyte-to-HDL-C Ratio

The results of our study showed that the ratio of absolute blood monocyte count to HDL-C concentration is associated with stenotic atherosclerosis of lower limb arteries: patients with values of this parameter above the median (>0.5) had a 2.7-fold higher probability of having the disease (OR = 2.7, *p* = 0.005), which is confirmed by results from other studies. Thus, Wei et al. demonstrated a significant increase in MHR with the progression of lower limb atherosclerosis, from 0.42 in patients without atherosclerosis to 0.60 in the group with severe stenosis [17]. According to the study by Song et al., the median MHR value was significantly higher in patients with type 2 diabetes and peripheral artery disease (0.37) compared to patients without peripheral atherosclerosis (0.29; *p* < 0.001) [10]. Conversely, in the study by Faizin et al., no statistically significant association was found between MHR and the severity of peripheral atherosclerosis. Although severe peripheral atherosclerosis was observed in 57.1% of patients with MHR above the median ( ≥14.5) compared to 42.9% in the group with MHR below the median, no statistically significant difference between the groups was detected (*p* = 0.1) [18].

### 3.3. Neutrophils and Neutrophil-to-Lymphocyte Ratio 

Patients with stenotic atherosclerosis of lower limb arteries detected at the first visit had significantly higher absolute neutrophil counts compared to patients in whom stenotic atherosclerosis was not detected at the second visit (4.2 [3.4; 5.4] × 10^9^/L vs. 3.6 [3.0; 4.6] × 10^9^/L, *p* = 0.04). This neutrophil elevation may reflect the activation of innate immunity and the development of a systemic inflammatory response in the atherosclerotic lesions of peripheral arteries. Unlike the literature data, in our study, the NLR itself was not independently associated with stenotic atherosclerosis of the lower limbs. However, it was found that the combination of a low NLR (below the median of 1.79) and Lp(a) concentration < 30 mg/dL was associated with a 60% reduction in peripheral atherosclerosis risk. Teperman et al., in a study of 733 patients, identified an independent association between elevated NLR and severe multilevel peripheral artery disease (OR 1.11; 1.03–1.19, *p* < 0.01), with this association persisting after the adjustment for classical risk factors [19]. Ye et al. demonstrated that patients in the highest NLR tertile had higher amputation rates (23.9%) and lower ankle–brachial index values compared to patients in the lowest tertile [20].

### 3.4. Neutrophil-to-HDL-C Ratio 

An NHR above the median in our study showed moderate association with atherosclerosis (OR = 2.0, *p* < 0.05). However, an NHR above the median (≥3.6) in the setting of elevated Lp(a) concentration (≥30 mg/dL) enhanced the association with peripheral atherosclerosis by more than 1.5-fold (OR 3.2; 1.3–8.2, *p* < 0.001). Studies show that NHR positively correlates with Gensini score (r = 0.287, *p* < 0.001) and is an independent predictor of coronary artery disease presence (OR = 1.163, 95% CI: 1.034–1.308, *p* = 0.012) [21]. The clinical significance of this parameter is confirmed by its superiority over isolated parameters: in patients with coronary artery disease, median NHR is significantly higher (3.7 vs. 3.2; *p* < 0.01), and a cut-off value ≥ 3.88 predicts severe coronary artery disease with 62.6% sensitivity and 66.2% specificity [21]. Studies show that in patients with type 2 DM diabetes and peripheral atherosclerosis, the median of this parameter was significantly higher than in patients without stenotic atherosclerosis (3.7 vs. 2.9, AUC 0.70; *p* < 0.001) [10].

### 3.5. Lymphocyte-to-Monocyte Ratio

Our study did not confirm a statistically significant independent association of LMR with stenotic atherosclerosis of the lower limbs. The combination of low LMR (<median 4.0) with elevated Lp(a) (≥30 mg/dL) was significantly more frequent in patients with stenotic atherosclerosis of lower limb arteries than in patients without peripheral atherosclerosis (21% vs. 9%; *p* < 0.05). However, when calculating odds ratios, the association with stenotic atherosclerosis of the lower limbs remained at the trend level (OR = 1.7; 95% CI: 0.9–3.2; *p* < 0.1). The most significant clinical data demonstrated that a decreased LMR is significantly associated with increased amputation risk after revascularization in patients with peripheral artery disease and diabetes mellitus: in the amputation group, mean LMR was 2.12 (95% CI: 1.67–4.40) versus 3.22 (95% CI: 2.22–5.08) in the group without amputations (*p* < 0.01), with a diagnostic threshold of LMR = 2.55 predicting amputation necessity with 56.2% sensitivity and 66.9% specificity [22]. Moreover, the LMR did not correlate with the number of affected vessels, indicating its specific role as a risk factor for adverse outcomes rather than anatomical disease severity [22].

### 3.6. Platelet-to-Lymphocyte Ratio 

In our study, a PLR below the median (<105.7) was associated with stenotic atherosclerosis of lower limb arteries with OR = 2.2, *p* < 0.05. Meanwhile, the combination of higher PLR ≥ 105.7 and Lp(a) concentration ≥ m 30 mg/dL demonstrated a significantly stronger association with stenotic atherosclerosis of lower limb arteries (OR 7.7; 95% CI 2.3–26.1, *p* = 0.0001) compared to the group of patients who did not have such a combination. The literature data on the association of the PLR with atherosclerosis remain contradictory. Ye et al. demonstrated that patients in the highest tertile of platelet-to-lymphocyte ratio had higher amputation rates (25.4%) and hospitalizations for acute lower limb ischemia within one year (73.5%) [23]. In the study by Selvaggio et al., a negative correlation between PLR and ankle–brachial index was found in elderly patients (ρ = −0.18, *p* < 0.001) [7]. However, in the study by Varim et al. [24], it was found that patients with critical carotid artery stenosis had a significantly lower PLR compared to patients with non-stenotic atherosclerosis (94.9 ± 60.3 vs. 162.5 ± 84.7, *p* < 0.001), with a PLR cut-off value of 117.1 showing high predictive ability (89% sensitivity and 68% specificity) for detecting critical stenosis.

### 3.7. Platelet-to-HDL-C Ratio 

The ratio of absolute blood platelet count to HDL-C concentration did not show significant differences as a single parameter in our study; however, the combination of this ratio above the median (203.0) and an elevated Lp(a) concentration (≥30 mg/dL) was significantly associated with the development of stenotic atherosclerosis of lower limb arteries (OR = 4.3; 95% CI: 1.6–11.4; *p* < 0.01). Zhang et al., in a large population-based study, showed that a PHR above 223.7 significantly increases the risk of stroke and cardiovascular mortality [11]. A large-scale multicenter Chinese study including 56,316 patients with a median follow-up of 5.32 years demonstrated that patients with a PHR above the median (≥233.8) and type 2 diabetes had the highest risk of all-cause mortality (19.2%) and cardiovascular mortality compared to other groups, with an adjusted risk of HR = 1.43 (95% CI 1.34–1.52) [25]. A study of coronary atherosclerosis severity involving 1721 patients revealed a linear relationship between the PHR and degree of coronary stenosis according to the Gensini scale, with patients in the highest PHR quartile demonstrating a 1.3-fold increased risk of severe lesions (95% CI 1.1–1.6), and this association was particularly pronounced in women (OR = 1.4) [26].

### 3.8. Lipoprotein(a)-to-HDL-C Ratio 

We found no information in the available literature describing studies of the lipoprotein(a)-to-HDL-cholesterol ratio [Lp(a)/HDL-C] in the context of atherosclerosis. However, our study data indicate a significant association of elevated Lp(a)/HDL-C ratio with peripheral atherosclerosis risk: adjusted odds ratio 2.5 (95% confidence interval: 1.3–4.9; *p* = 0.008). In our view, this ratio represents a promising parameter that integrates information about genetically determined atherogenic risk and endogenous anti-atherogenic protection. This parameter may find application in composite lipid indices for more accurate cardiovascular risk stratification; however, further prospective studies are needed to validate the clinical utility of this ratio and establish reference values for different populations.

### 3.9. Composite Index [0.3 × Lp(a) Concentration (mg/dL) + 0.7 × Absolute Monocyte Count (×10^9^/L)]

When comparing our composite index (AUC = 0.63) with other PAD predictors from the literature, various levels of diagnostic accuracy are observed. Song et al. [10] reported higher AUC values for individual inflammatory biomarkers in patients with type 2 diabetes and PAD: NHR (AUC = 0.70), SIRI (AUC = 0.71), MHR (AUC = 0.68), AISI (AUC = 0.67), SII (AUC = 0.64), and PHR (AUC = 0.606), with the combined NHR + SIRI model showing the best performance with AUC = 0.733. Ye et al. [20] demonstrated AUC values of 0.73 for NLR and 0.67 for PLR in predicting the progression of chronic lower limb ischemia. However, it is important to note that our composite index significantly outperforms the MHR from the study by Irawan et al. [18], which showed an AUC of only 0.55 for predicting LEAD severity.

Despite the moderate AUC value, our composite index has important clinical advantages. First, it incorporates Lp(a)—an established independent risk factor for atherosclerosis that is not considered in other inflammatory indices—providing pathophysiological rationale through the combination of an inflammatory component (monocytes) and an atherogenic component (Lp(a)). Second, our data demonstrate pronounced prognostic value regarding CLLI development in patients with PAD when performing Cox regression, where the risk of CLLI development in patients with index values above the median was 80% higher than in patients with composite index values below the median, regardless of arterial hypertension, type 2 diabetes mellitus, and sex.

## 4. Materials and Methods

The study included 361 patients: 238 patients (65.9%) had stenotic atherosclerosis of lower limb arteries detected at the first visit (Group 1) and 123 patients (34.1%) had no stenotic atherosclerosis of the lower limbs at the first visit. The second visit was conducted in 281 patients: 158 patients with atherosclerosis of lower limbs verified at the first visit (from Group 1), 32 patients who initially had no stenotic atherosclerosis of lower limbs but were diagnosed at the second visit (Group 2), and 91 patients in whom stenotic atherosclerosis of lower limb arteries was not detected either at the first or second visit comprised Group 3. The exclusion criteria from the study were diagnosed malignancies, autoimmune, inflammatory, or infectious diseases. Only participants who were free of these conditions were invited to return.

**Figure 9:**
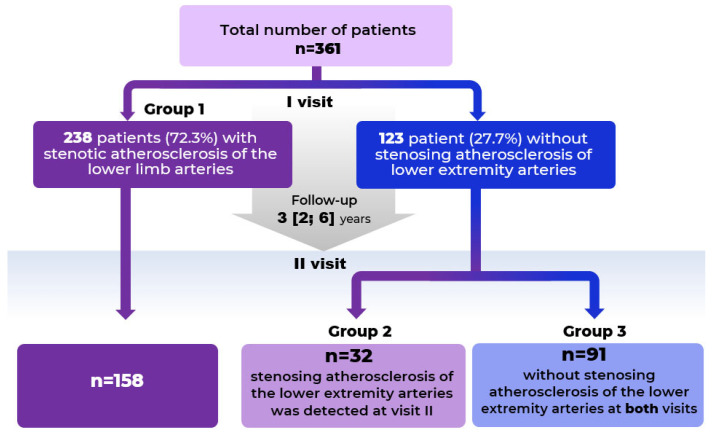
Study design.

The observation period between the first and second visit was 3 [2, 6] years (Figure 9). The study was conducted in accordance with good clinical practice and the Declaration of Helsinki. The study protocol was approved by the local ethics committee (No. 251, 25 November 2019) and written informed consent was obtained from all participating patients before inclusion in this study.

The diagnosis of atherosclerosis of lower limb arteries was performed using duplex ultrasonography [27]. The examination included an assessment of vascular morphology (visualization of atherosclerotic plaques, measurement of intima-media thickness) and analysis of hemodynamic parameters (spectral Doppler blood flow) in standard segments: aorto-iliac, femoral, popliteal, and tibial arteries. Stenosis was considered hemodynamically significant (≥50%) when the following criteria were identified: increased peak systolic velocity (PSV) in the area of suspected narrowing compared to the proximal unchanged segment (PSV ratio ≥ 2.0), presence of turbulent flow distal to the stenosis, and changes in the spectral waveform distal to the lesion site. DUS of lower limb arteries was performed using the iU-22 and Epic-5 ultrasound systems (Philips, Amsterdam, The Netherlands) with a 3–9 MHz linear transducer.

Lipid concentrations total cholesterol (TC), triglycerides (TG), HDL-C were determined by the enzymatic colorimetric method using an Architect C-8000 (Abbott, Abbott Park, IL, USA) biochemical analyzer. Quality control was performed using Precinorm and Precipath control sera (Roche Diagnostics, Basel, Switzerland). Low-density lipoprotein cholesterol (LDL-C) was calculated using the Friedewald formula.

Lipoprotein(a) [Lp(a)] concentration was determined by an interlaboratory enzyme-linked immunosorbent assay using monospecific polyclonal anti-human Lp(a) antibodies, as previously described [28]. The sensitivity of the method was 0.2 mg/dL, the intra-plate and inter-experiment variation coefficients were 3.8% and 9.8%, respectively, in the Lp(a) concentration ranging from 5 to 190 mg/dL. The method was validated with two kits: TintElize Lp(a) (Biopool AB, Umea, Sweden) and Immunozym Lp(a) (Progen Biotechnik GmbH, Heidelberg, Germany). The control serums (Technoclone, Vienna, Austria) and (DiaSys Diagnostic Systems GmbH, Holzheim, Germany) which were approved by the International Federation of Clinical Chemistry were used to validate the accuracy of the ELISA [29]. Also, the regression analysis showed high data consistency (r = 0.99, *p* < 0.001) of the intralaboratory method with the method of a latex enhancement turbidimetric assay for the Abbott Architect 4000 biochemical analyzer (Abbott) when determined the Lp(a) concentration in 300 non-frozen serum samples. 

C-reactive protein was measured in 136 patients from Groups 1 and 2. Serum C-reactive protein (CRP) concentration was determined by the immunoturbidimetric method using an Abbott Architect 4000 biochemical analyzer (Abbott).

Based on blood count analysis, composite indices were calculated: LMR as the ratio of absolute lymphocyte count to monocytes, NLR as the ratio of absolute neutrophils count to lymphocytes, and PLR as the ratio of absolute platelets count to lymphocytes Additionally, the following ratios were determined: monocytes-to-HDL-C (MHR), neutrophils-to-HDL-C (NHR), Lp(a)-to-HDL-C, platelets-to-HDL-C (PHR), and monocytes-to-Lp(a).

The composite index was calculated as follows: both Lp(a) and monocytes demonstrated statistically significant correlations with atherosclerosis outcomes (Lp(a): r = 0.191, *p* < 0.001; monocytes: ρ = 0.165, *p* = 0.003) and were combined into a composite biomarker. Prior to combination, both variables were standardized using z-score transformation (X_standardized = (X - μ) / σ) to account for different units of measurement, with standardization parameters: Lp(a) μ = 42.9 mg/dL, σ = 52.7 mg/dL; monocytes μ = 0.63 × 10^9^/L, σ = 0.56 × 10^9^/L. Optimal weight coefficients were determined through the systematic evaluation of nine weight combinations (ranging from 0% to 100% with 10% increments) using the area under the ROC curve (AUC) as the selection criterion. The combination of 30% Lp(a) + 70% monocytes achieved the highest predictive performance (AUC = 0.6164) and was selected for the final model. Thus, the composite index was calculated using the formula: Composite Index = [0.3 × Lp(a) concentration (mg/dL) + 0.7 × absolute monocyte count (×10^9^/L)].

Quantitative variables were tested for normality; the Shapiro–Wilk test was used for n < 50, and the Kolmogorov–Smirnov test for n ≥ 50. Data with normal distribution were described using arithmetic mean (M), standard deviation (SD), and 95% confidence interval (95% CI) for the mean. For non-normally distributed data, median (Me) and interquartile range (Q1–Q3) were presented. Categorical data were described using absolute values and percentages with 95% CI calculated by the Clopper–Pearson method. For the comparison of three or more groups by quantitative variables, when normal distribution was present in all groups, one-way analysis of variance (ANOVA) with Tukey’s post hoc comparisons was used (contingent upon homogeneity of variance); for deviations from normality, the Kruskal–Wallis test with Dunn’s post hoc comparison and Holm correction was applied.

For several parameters, the NNH (Number Needed to Harm) was calculated—a statistical measure that indicates the number of patients who need to be exposed to a risk factor for one of them to develop an adverse outcome. When calculating the odds ratios for the development of stenotic atherosclerosis of the lower limbs in the presence of two combined study parameters, the Common Reference Group principle was employed. In this approach, the reference group included all possible combinations of the two parameters except for the investigated combination. This methodology ensures a methodologically sound assessment of the effects of specific risk factor combinations and eliminates the problem of shifting reference groups. 

Statistical evaluation included ROC analysis to determine the discriminating ability of the model and for the determination of the optimal cut-off value. A comparison of percentages in contingency tables was performed using Pearson’s χ^2^ test; post hoc pairwise comparisons also used χ^2^ with Holm correction. Statistical significance was established at *p* < 0.05.

## 5. Conclusions

The results of our study confirm the important role of systemic inflammation in the pathogenesis of lower-limb artery atherosclerosis, particularly in patients with elevated Lp(a) levels. An important advantage of cellular indices is their accessibility for routine clinical use, as the calculation of these parameters requires no additional financial cost and can be performed based on standard laboratory investigations, making them attractive tools for risk stratification and the personalized management of patients with atherosclerosis. Further prospective studies are needed to validate these findings and determine the optimal cut-off values for different patient populations.

## 6. Study Limitations

1. The design of the second visit involved selective participant selection: not all participants from the first visit were invited to the second visit. Of the 238 patients with initially diagnosed PAD, 158 were enrolled on the second visit after responding to the invitation and meeting no exclusion criteria. This subgroup for re-examination was formed randomly and was not based on any specific clinical or laboratory parameters. The sample size for the stenotic atherosclerosis group (n = 158) was determined based on statistical power calculations. All 123 patients without stenotic atherosclerosis of the lower limb arteries diagnosed at visit one were invited to the second visit.
2. Despite the fact that the exclusion criteria from the study were diagnosed malignancies, and autoimmune, inflammatory or infectious diseases, we recognize that inflammatory markers are subject to internal variability and may depend on various factors, including subclinical infectious processes, which could affect the results.
3. The relatively small sample size of patients with atherosclerosis first detected at visit two may limit the statistical power of the study when analyzing weak associations between certain markers and disease risk.
4. The absence of data on smoking history and the degree of arterial hypertension control among participants limits the ability to assess the contribution of this risk factor to disease development.
5. The prognostic stability of the developed composite index in various clinical subgroups was not evaluated in this study, which limits its potential for widespread clinical application. Multicenter prospective cohort studies are needed to validate the index in different patient populations, optimize threshold values, and assess its stability in different clinical scenarios.

## Figures and Tables

**Table 1:** Characteristics of patients depending on the presence or absence of stenotic atherosclerosis of the lower limb arteries (first visit).

Indicators	Stenotic Atherosclerosis at Visit I n = 238 (Group 1)	Stenotic Atherosclerosis at Visit II n = 32 (Group 2)	Stenotic Atherosclerosis Is Not Detected at Both Visits n = 91 (Group 3)	*p*
Male sex	194 (81.5%)	25 (78.1%)	59 (64.8%)	0.006 * *p*_3−1_ = 0.004
CAD	192 (80.7%)	15 (46.9%)	38 (41.8%)	<0.001 * *p*_3−1_ <0.001 *p*_1−2_ < 0.001
Age	62.0 [54.0; 68.0]	59.0 [50.8; 70.5]	56.0 [51.0; 65.0]	0.1
MI	122 (51.3%)	12 (37.5%)	18 (19.8%)	<0.001 * *p*_3−1_ < 0.001
PCI	128 (53.8%)	11 (34.4%)	24 (26.4%)	<0.001 * *p*_3−1_ < 0.001
CABG	28 (11.8%)	1 (3.1%)	1 (1.1%)	0.004 * *p*_3−1_ = 0.007
Arterial hypertension	189 (79.4%)	21 (65.6%)	73 (80.2%)	0.2
Obesity	171 (71.8%)	30 (93.8%)	70 (76.9%)	0.02 * *p*_1−2_ = 0.02
Type 2 DM	50 (21.0%)	4 (12.5%)	12 (13.2%)	0.2
Statins	117 (49.2)	10 (31.2%)	45 (49.5)	0.1
The dose of statins equivalent to atorvastatin	40.0 [20.0; 60.0]	40.0 [20.0; 40.0]	40.0 [20.0; 60.0]	0.9
**Complete Blood Count**				
Leukocytes, 10^9^/L	6.8 [5.0; 9.0]	6.3 [4.7; 7.8]	6.0 [5.0; 7.7]	0.1
Lymphocytes, 10^9^/L	2.3 [1.8; 2.7]	2.2 [1.9; 2.5]	2.1 [1.8; 2.6]	0.5
Lymphocytes, %	30.4 ± 8.2	32.2 ± 8.5	33.26 ± 10.33	0.09
Monocytes, 10^9^/L	0.58 [0.45; 0.71]	0.57 [0.38; 0.70]	0.52 [0.40; 0.60]	0.05
Monocytes, %	7.92 ± 2.01	7.75 ± 2.28	7.79 ± 2.07	0.9
Neutrophils, 10^9^/L	4.2 [3.4; 5.4]	3.9 [3.0; 4.9]	3.6 [3.0; 4.6]	0.04 * *p*_1−3_ = 0.04
Neutrophils, %	57.9 ± 8.6	56.3 ± 8.6	56.0 ± 10.4	0.4
Platelets, 10^9^/L	217.5 [187.5; 262.5]	241.0 [217.0; 279.0]	230.0 [199.0; 284.0]	0.1
**Biochemical parameters**				
CRP, mg/L	1.1 [0.4; 2.4]	0.7 [0.3; 1.1]	0.4 [0.1; 0.7]	0.01 * *p*_1−3_ = 0.02
TC, mmol/L	4.9 [3.9; 6.1]	5.7 [4.6; 6.4]	5.4 [4.4; 6.1]	0.2
HDL-C, mmol/L	1.07 [0.89; 1.26]	1.40 [1.02; 1.67]	1.15 [1.0; 1.5]	0.003 * *p*_1−3_ = 0.02 *p*_2−1_ = 0.03
LDL-C, mmol/L	2.9 [2.1; 4.0]	3.50 [2.34; 4.11]	3.2 [2.3; 4.2]	0.2
TG, mmol/L	1.51 [1.19; 2.18]	1.33 [0.99; 1.95]	1.70 [1.16; 2.29]	0.4
Lp(a), mg/dL	20.2 [6.7; 88.5]	16.7 [7.5; 58.7]	12.1 [5.2; 26.8]	0.007 * *p*_1−3_ = 0.005
Lp(a) ≥ 30 mg/dL	103 (43.3%)	13 (40.6%)	22 (24.2%)	0.006 * *p*_3−1_ = 0.004
**Investigated Indices**				
LMR	3.9 [3.1; 4.8]	4.3 [3.0; 5.4]	4.3 [3.5; 5.1]	0.3
NLR	1.8 [1.5; 2.5]	1.6 [1.3; 2.4]	1.7 [1.4; 2.3]	0.2
MHR	0.54 [0.40; 0.73]	0.46 [0.25; 0.66]	0.39 [0.30; 0.55]	0.002 * *p*_1−3_ = 0.002
NHR	3.9 [2.9; 5.5]	3.2 [1.9; 3.96]	3.3 [2.3; 4.1]	0.002 * *p*_1−3_ = 0.007
Lp(a)/HDL-C	20.9 [6.2; 71.8]	11.5 [5.6; 54.3]	8.8 [4.4; 21.4]	0.003 * *p*_1−3_ = 0.002
PHR	207.2 [162.0; 260.8]	200.9 [154.6; 246.1]	192.7 [154.6; 255.6]	0.6
PLR	98.1 [74.3; 124.1]	111.8 [92.2; 122.0]	115.2 [90.4; 147.8]	0.03 * *p*_1−3_ = 0.03

Note: * *p*-trend for all three groups. CAD = coronary artery disease; PCI = percutaneous coronary intervention; CABG = coronary artery bypass graft; Type 2 DM = type 2 diabetes mellitus; CRP = C-reactive protein; LMR = lymphocyte–monocyte ratio; NLR = neutrophil–lymphocyte ratio; Lp(a) = lipoprotein(a); HDL-C = high-density lipoprotein cholesterol; LDL-C = low-density lipoprotein cholesterol; TC = total cholesterol; TG = triglycerides; MHR = monocyte-to-HDL-cholesterol ratio; NLR = neutrophils and neutrophil-to-lymphocyte ratio; NHR = neutrophil-to-HDL-cholesterol ratio; PLR = platelet-to-lymphocyte ratio; PHR = platelet-to-HDL-cholesterol ratio. Quantitative parameters: presented as median with interquartile range [Q1; Q3] or mean ± standard deviation. Categorical parameters: n [11].

**Table 2:** Characteristics of patients depending on the presence or absence of stenotic atherosclerosis of the lower limb arteries (second visit).

Indicators	Stenotic Atherosclerosis at Visit I (n = 158) Group 1	Stenotic Atherosclerosis is Not Detected at Both Visits (n = 91) Group 3	Stenotic Atherosclerosis at Visit II (n = 32) Group 2	*p*
Age, years	62.5 [52.0; 67.8]	62.0 [55.0; 69.0]	63.0 [54.3; 71.8]	0.4 *
Male sex	130 (82.3%)	59 (64.8%)	25 (78.1%)	0.008 *p*_1−3_ = 0.006
CAD	145 (95.4%)	61 (79.2%)	27 (90.0%)	<0.001 * *p*_1−3_ < 0.001
MI	111 (70.3%)	35 (50.0%)	17 (63.0%)	0.01 * *p*_1−3_ = 0.01
PCI	76 (59.4%)	31 (55.4%)	13 (72.2%)	0.4 *
CABG	6 (4.7%)	7 (12.5%)	1 (5.6%)	0.1 *
Arterial hypertension	112 (87.5%)	48 (85.7%)	16 (88.9%)	0.9 *
Obesity	42 (32.8%)	14 (25.0%)	7 (38.9%)	0.4 *
Type 2 DM	30 (23.4%)	14 (25.0%)	6 (33.3%)	0.6 *
**Complete Blood Count**				
Leukocytes, 10^9^/L	6.6 [5.2; 8.2]	6.9 [5.6; 8.6]	6.8 [5.6; 7.8]	0.7 *
Lymphocytes, 10^9^/L	2.1 [1.7; 2.6]	2.3 [1.8; 2.8]	2.2 [1.9; 2.5]	0.5 *
Lymphocytes, %	30.8 [25.9; 35.8]	33.9 [28.4; 38.9]	31.0 [25.3; 34.8]	0.02 * *p*_1−3_ = 0.04
Monocytes, 10^9^/L	0.6 [0.4; 0.7]	0.6 [0.5; 0.7]	0.6 [0.5; 0.7]	0.8 *
Monocytes, %	7.8 [6.6; 9.2]	7.9 [6.5; 9.3]	8.2 [7.1; 10.1]	0.8 *
Neutrophils, 10^9^/L	4.0 [3.0; 5.3]	3.9 [3.0; 4.6]	3.9 [3.3; 4.9]	0.6 *
Neutrophils, %	56.3 [51.3; 62.8]	54.8 [51.1; 58.9]	56.9 [50.4; 63.5]	0.2 *
Platelets, 10^9^/L	231.5 [192.8; 269.5]	221.0 [199.0; 274.0]	227.5 [199.5; 262.3]	0.9 *
**Investigated Indices**				
LMR	4.0 [3.1; 4.7]	4.0 [3.2; 5.0]	3.6 [3.0; 4.8]	0.6 *
NLR	1.8 [1.4; 2.4]	1.6 [1.3; 2.2]	1.9 [1.4; 2.5]	0.07 *
MHR	0.4 [0.3; 0.6]	0.5 [0.3; 0.6]	0.6 [0.4; 0.7]	0.5 *
NHR	3.2 [2.4; 4.5]	3.5 [2.7; 4.6]	3.8 [3.1; 4.1]	0.7 *
Lp(a)/HDL-C	17.8 [5.1; 75.2]	11.4 [5.7; 27.3]	39.5 [10.4; 97.8]	0.2 *
PHR	199.4 [158.1; 247.8]	192.6 [173.3; 239.5]	177.3 [164.0; 239.0]	0.8 *
PLR	102.4 [87.6; 135.7]	101.7 [79.4; 127.5]	94.1 [83.9; 120.4]	0.3 *
**Biochemical parameters**				
TC, mmol/L	4.0 [3.4; 5.1]	4.1 [3.5; 5.1]	4.0 [3.2; 5.0]	0.9 *
HDL-C, mmol/L	1.1 [0.9; 1.4]	1.1 [0.9; 1.3]	1.0 [0.9; 1.3]	0.7 *
LDL-C, mmol/L	2.1 [1.6; 2.9]	2.0 [1.6; 3.0]	2.5 [1.6; 2.9]	0.9 *
TG, mmol/L	1.3 [1.0; 1.8]	1.6 [1.2; 2.3]	1.5 [1.2; 2.5]	0.08 *
CRP	1.5 [0.4; 7.7]	4.6 [0.5; 8.1]	3.8 [1.4; 10.0]	0.5 *
Lp(a), mg/dL	27.2 [6.9; 103.2]	12.1 [5.2; 26.8]	16.7 [7.5; 58.7]	<0.001 * *p*_1−3_ < 0.001
Lp(a) ≥ 30 mg/dL	75 (47.5%)	22 (24.2%)	13 (40.6%)	0.001 * *p*_1−3_ < 0.001
Lp(a) ≥ 50 mg/dL	59 (38.1%)	14 (15.6%)	9 (28.1%)	<0.001 * *p*_1−3_ < 0.001

Note: * *p*-trend for all three groups. CAD = coronary artery disease; PCI = percutaneous coronary intervention; CABG = coronary artery bypass graft; Type 2 DM = type 2 diabetes mellitus; CRP = C-reactive protein; LMR = lymphocyte–monocyte ratio; NLR = neutrophil–lymphocyte ratio; Lp(a) = lipoprotein(a); HDL-C = high-density lipoprotein cholesterol; LDL-C = low-density lipoprotein cholesterol; TC = total cholesterol; TG = triglycerides; MHR = monocyte-to-HDL-cholesterol ratio; NLR = neutrophils and neutrophil-to-lymphocyte ratio; NHR = neutrophil-to-HDL-cholesterol ratio; PLR = platelet-to-lymphocyte ratio; PHR = platelet-to-HDL-cholesterol ratio. Quantitative indicators: presented as median with interquartile range [Q1; Q3] or mean ± standard deviation. Categorical indicators: n [11].

## Data Availability

The data presented in this study are available upon request from the corresponding author.

## Abbreviations

The following abbreviations are used in this manuscript:

CABG	Coronary artery bypass graft	MHR	Monocyte-to-HDL-cholesterol ratio
CAD	Coronary artery disease	MI	Myocardial infarction
CI	Confidence interval	NNH	Number needed to harm
CLLI	Chronic lower limb ischemia	NHR	Neutrophil-to-HDL-cholesterol ratio
CRP	C-reactive protein	NLR	Neutrophil-to-lymphocyte ratio
DM	Diabetes mellitus	OR	Odds ratio
DUS	Duplex ultrasonography	PAD	Peripheral artery disease
HDL-C	High-density lipoprotein cholesterol	PCI	Percutaneous coronary intervention
HR	Hazard ratio	PHR	Platelet-to-HDL-cholesterol ratio
IQR	Interquartile range	PLR	Platelet-to-lymphocyte ratio
LDL-C	Low-density lipoprotein cholesterol	PSV	Peak systolic velocity
LMR	Lymphocyte-to-monocyte ratio	RR	Relative risk
Lp(a)	Lipoprotein(a)	TC	Total cholesterol
LLA	Lower limb arteries	TG	Triglycerides
Me	Median		
